# Histopathological and immunohistochemical profiles of pulp tissues in immature dogs’ teeth to two recently introduced pulpotomy materials

**DOI:** 10.1007/s00784-023-04915-5

**Published:** 2023-02-13

**Authors:** Mai Mohamed, Ahmed Abdel Rahman Hashem, Maram Farouk Obeid, Ashraf Abu-Seida

**Affiliations:** 1 Faculty of Dentistry, Ain Shams University, Cairo, Egypt; 2 Faculty of Veterinary Medicine, Cairo University, Cairo, Egypt

**Keywords:** Dentin bridge, Mineral trioxide aggregate, Pulpine mineral, Pulpine NE, Pulpotomy

## Abstract

**Objective:**

The pulpal response to Hoffmann’s Pulpine mineral (PMIN) and Pulpine NE (PNE) was compared to mineral trioxide aggregate (MTA) when used as pulpotomy materials in immature permanent teeth in dogs.

**Materials and methods:**

Immature premolars were randomly divided according to the observation period into three equal groups (*n* = 24) (10 days, 30 days, and 90 days) then furtherly subdivided into 3 subgroups according to the material used. Histopathological analysis regarding inflammatory cell infiltration and dentin bridge (DB) formation was done. Immunohistochemical analysis was performed using osteopontin marker.

**Results:**

The results showed that after 90 days, both MTA and PMIN subgroups had 100% complete thick DB without inflammation in 87.5% of the samples, while the PNE subgroup failed to form DB in 37.5% of the samples and 50% of samples showed thin initial DB with heavy inflammation in 62.5% of the samples. There was no significant difference between MTA and PMIN, while there was a statistically significant difference between PNE and the two other subgroups in DB formation and inflammatory cell infiltration (*P* > 0.05). After 90 days, MTA showed the highest mean value of osteopontin positive fraction area followed by PMIN without statistically significant differences, while the least value was recorded in PNE subgroup with statistically significant difference with the remaining subgroups (*P* < 0.05).

**Conclusion:**

PMIN is a promising alternative to MTA when used for pulpotomy.

**Clinical relevance:**

Vital pulp therapy in immature teeth can be done using PMIN as an alternative to MTA.

## Introduction

With the era of conservative dentistry and minimally invasive tactics in endodontic treatment, vital pulp therapy is recommended whenever the exposed pulp shows signs of reversible pulpitis [1]. Several techniques are applied for this; a pulpotomy procedure is one in which the inflamed coronal pulp is removed then capped with a pulpotomy material [2]. The main aim of this procedure is to help the formation of dentine bridge (DB) in order to guard against microbial infection and to preserve the pulpal tissue health [3]. It is planned to avert root canal therapy or at best delay till complete root maturation in teeth with traumatic injuries, deep caries, or mechanical exposures [2]. It relies on the superior biological ability of dental pulp to heal; named reparative dentinogenesis [4]^.^

This whole process depends mainly upon the selection of an ideal pulpotomy material. Ideal pulpotomy materials should have efficient antibacterial action, induction of tissue healing, biocompatibility, and sealing ability [5].

Varieties are available under which the formation of DB occurs, but MTA have proved to owe lesser inflammatory reaction and obvious development of the desired DB gaining the best results [5]. Regrettably, MTA showed discoloration, long setting time, and poor handling characteristics [6]. Thus, substitutes to MTA are presented in the market and grab attention.

With the present call back to nature, a paradigm change from medicaments to natural therapies is prevalent. The medical usage of products made by honeybees is widely spreading nowadays. This involves the usage of honey, pollen, royal jelly, and propolis (PS) [7]. The PS extract has high concentrations of the flavonoids, aromatic acids, and esters which have antimicrobial, and anti-inflammatory properties [8]. Additionally, the ability of PS to form hard tissue promoting regeneration when used as a vital pulp therapy medicament agent was highlighted by many researchers [9–13]. Hoffmann dental manufacture (Hoffmann Dental Manufaktur, HDM, Berlin, Germany) used PS as the main ingredient in the liquid of two new materials substituting MTA. The first product is PMIN which is composed of 70% hydroxyapatite (HAP) implanted in the composite of calcium hydroxide and PS [14]. HAP is a calcium phosphate biomaterial previously used as pulp capping material due to its biocompatibility and osteoconductivity [15]. The other eugenol-free biocompatible MTA substitute is PNE which contains PS, zinc compounds, and calcium compounds [16]. The last reacts with the carbon dioxide in the tissue, and forms calcite crystals initiating the calcification process [17].

The rationale of our present study was to compare histologically and immunohistochemistry the new Hoffmann’s PMIN and PNE to MTA as pulpotomy materials in immature permanent teeth in dogs. Our null hypothesis was that no difference in the histologic outcome of pulpotomy between tested materials, measured as calcific barrier continuity and the thickness of DB as well as inflammatory cells infiltration.

## Materials and methods

This prospective animal study was approved by the Research Ethics Committee at our university (END 16-18D/7–2017) and followed up the Animal Research: Reporting of In Vivo Experiments criteria based on the protocol established by ISO 7405:2018.

### Group classification and randomization

Sample size calculations were established on the null hypothesis of being no difference in the histologic results of pulpotomy between the tested materials, measured as calcific barrier continuity and the thickness of DB as well as inflammatory cells infiltration. The study was designed to detect a minimum difference of 20% in the quantity of continuous calcified barriers between materials, considering 80% beta-power and a significance level of 0.05 (alpha error) and effect size 0.7. Power calculations determined a total of 63 samples, 21 per group (*n* = 7 for subgroup) (10, 30, and 90 days). This was increased to sample size of 8 to account for a 10% dropout rate (potential animal death or discrepancies during the histopathological process), resulting in a total sample size of 72 teeth, 24 per group (*n* = 8 for subgroup) [18]. Six mongrel dogs (4–6 months age) of both sexes were randomly divided according to the post-treatment observation period into three main groups of 2 dogs (24 teeth each): GP1: 10 days, GP2: 30 days, and GP3: 90 days. Each main group was further subdivided into three subgroups (8 teeth each) according to the applied pulpotomy material; PNE, PMIN, and MTA subgroups. All subgroups were represented in each quadrant in the dog’s mouth randomly.

### Surgical procedure

For anesthesia, atropine sulfate (Atropine sulphate®, ADWIA CO., Egypt) was injected at 0.1 mg/kg subcutaneously, followed by 1 mg/kg intravenous injection of Xylazine HCl (Xylaject®, ADWIA, CO., Egypt). Ketamine HCl (Keiran®, EIMIC CO., Egypt), 5 mg/kg, was given intravenously via a 20-gauge IV cannula to produce general anesthesia followed by 25 mg/kg of 2.5% thiopental sodium (thiopental sodium®, EIPICO, Egypt) given as a dose to effect intravenously.

The surfaces of the teeth were cleansed with povidone-iodine solution and were adequately isolated with sterilized cotton cylinders. A mouth gage was applied to separate the jaws. A typical access cavity was prepared in three premolars with no. 2 rose head carbide bur under copious saline irrigation in each quadrant. Then, complete deroofing was carried out using diamond stone. As the teeth were vital with normal pulp, the bleeding was controlled by pressing a cotton pellet until the physiologic hemostasis occurred.

The pulp was directly capped according to the matching subgroup and following the manufacturer’s instructions by blending the powder with the liquid supplied with each agent to get a homogenous mix. In the MTA subgroup, the exposure was capped by ProRoot MTA (Dentsply Tulsa, Tulsa, OK, USA). In the PMIN subgroup, the exposed pulp was capped by PMIN; one spoon of the powder was mixed with three drops of the supplied liquid. In the PNE subgroup, the exposure site was capped by PNE; one spoon of the powder was mixed with four drops of liquid. All dogs were handled by a single operator. Carprofen tablets (Rimadyl®, Zoetis, USA) at a dose of 4.4 mg/kg were given once orally for 5 days to control the post-operative pain.

### Methods of evaluation

#### Histopathological evaluation

According to the main groups, the dogs were euthanized at the end of the observation periods by an overdose of thiopental sodium administered intravenously. Premolars with the surrounding bone were dissected, preserved for 2 weeks in a 10% buffered formalin solution, and subsequently decalcified for 120 days in a 17% EDTA solution. Decalcified specimens were dehydrated in 70% ethanol, encased in paraffin blocks, micro-sections were cut buccolingually at a thickness of 1 mm, and stained with hematoxylin and eosin dye. To eliminate bias, encoded specimens were used all over the investigation. By a high-resolution camera (Carl Zeiss Imager D1 Axio, Goettingen, Germany) connected to an optical microscope (Axio Cam MRc5; Carl Zeiss Microimaging, Thornwood, NY) the following histopathological findings were evaluated according to Nowicka et al. [19] and Santos et al. [20] (Table 1) with slight modifications.

**Table 1:** The scores for histopathological evaluation of dentine bridge formation and capped pulp inflammation

Calcific barrier continuity	
Score	Explanation
1	Complete bridge
2	Partial/incomplete bridge reaching more than one half of the exposure
3	Initial bridge reaching less than one half of the exposure
4	No bridge
Dentine bridge thickness	
1	Thick: > 0.25 mm
2	Moderate: 0.1–0.25 mm
3	Thin: < 0.1 mm
4	Absent
Inflammatory cell infiltration	
1	Absence of inflammatory cells
2	Mild inflammatory cells infiltration
3	Moderate inflammatory cells infiltration
4	Heavy inflammatory cells infiltration

Image-J analysis software was used to measure all following parameters:

1. Dentine bridge formation: at × 10 the formed bridge (if present) was evaluated whether initial, partial, or complete.
2. Dentine bridge thickness: the thickness was measured at the bridge’s thickest, thinnest, and middle points. A representative slide’s mean of three lengths was generated then classified whether DB > 0.25 mm (thick), 0.1–0.25 mm (moderate), or < 0.1 mm (thin) or absent.
3. Inflammatory cells infiltrations in the dental pulp: at × 40, four representative areas from each specimen with well-preserved tissue and extensive infiltration of inflammatory cells were examined then categorized to Absence; with no inflammatory cells infiltration, Mild; when the inflammatory cells are present close to the area of pulp exposure; Moderate, when inflammatory cells are present in part of the coronal pulp; and Heavy infiltration; when the inflammatory cells are present throughout the entire coronal pulp. All analyses were performed separately in a blinded manner by two calibrated investigators.

#### Immunohistochemical evaluation

Immunohistochemistry analysis for osteopontin (OPN) was performed for group 3 only (i.e., after 90 days). Briefly, histological sections were incubated with primary antibodies: goat anti-OPN (SC-10593, Santa Cruz Biotechnology, Santa Cruz, CA, USA), then incubated with biotinylated secondary antibody and subsequently treated with conjugated streptavidin/HRP (Universal Dako Labeled HRP Streptavidin–Biotin Kit, Dako Laboratories, Carpinteria, CA, USA). Visualization was done with 3,3′-diaminobenzidine tetrahydrochloride (DAB Chromogen Kit, Dako Laboratories) as a chromogen. For the negative control group: the primary antibody was substituted by nonspecific serum of the same dilution then completed as mentioned.

OPN was assessed in the connective tissue near the material in an area of 66.55 × 10^3^ um^2^. Immunolabeling was specified as the appeared brown color areas. An automated measuring of the percent of the area of positive reaction was done using the image analyzer software (Leica Qwin software version 3 7.0, Switzerland) for images obtained with the aid of a high-resolution camera (Carl Zeiss Imager D1 Axio, Goettingen, Germany) connected to an ocular microscope (Axio Cam MRc5; Carl Zeiss Microimaging, Thornwood, NY) (× 20) [21]^.^

### Statistical analysis

The statistical analysis was carried out using the Statistical Package for the Social Sciences (SPSS 25, IBM, USA). The chi-square test was used to compare scores of dentin bridge formation and inflammatory cell infiltration between the groups, with adjusted residuals utilized to find the significant one. The Kruskal–Wallis test was used to evaluate scores of DB thickness between the groups and followed by the Mann–Whitney *U* test for pairwise comparisons with Dunn-Bonferroni adjustment. For intragroup comparison, the chi-square test was used. Regarding immunohistochemical results, ANOVA test was utilized and followed by post hoc test. At *P* < 0.05, the differences were statistically significant. The values were calculated by a single investigator who was unaware of the materials’ identities.

## Results

All dogs showed no signs of distress at the end of the planned observation period, and all teeth were histologically examined.

### Histopathological findings

The scores obtained for each material in terms of calcific barrier continuity, thickness of DB, and pulp tissue inflammation over the observation periods are shown in Table 2.

**Table 2:** The scores (%) of the tested materials at different observation periods regarding the evaluated histopathological findings

Calcific barrier continuity													
Score (%)	Observation period	10 days				30 days				90 days			
	Material	MTA^a^	PMIN^a^	PNE^b^	*P*-value	MTA^a^	PMIN^a^	PNE^b^	*P*-value	MTA^a^	PMIN^a^	PNE^b^	*P*-value
1	Complete	25	50	0	0.039*	100	100	0	0.014*	100	100	0	0.014*
2	Partial/incomplete (> half exposure)	37.5	37.5	12.5		0	0	12.5		0	0	12.5	
3	Initial (< half exposure)	37.5	12.5	37.5		0	0	50		0	0	50	
4	No bridge	0	0	50		0	0	37.5		0	0	37.5	
Dentine bridge thickness													
1	Thick: > 0.25 mm	25	50	0	0.001*	87.5	87.5	12.5	0.001*	87.5	100	0	0.034*
2	Moderate: 0.1–0.25 mm	75	50	25		12.5	12.5	50		12.5	0	12.5	
3	Thin: < 0.1 mm	0	0	25		0	0	0		0	0	50	
4	Absent	0	0	50		0	0	37.5		0	0	37.5	
Inflammatory cell infiltration													
1	Absence of inflammatory cells	50	50	0	0.001*	75	75	0	0.005*	87.5	87.5	0	0.002*
2	Mild inflammatory cells	50	50	0		25	12.5	12.5		12.5	12.5	12.5	
3	Moderate inflammatory cells	0	0	50		0	12.5	25		0	0	25	
4	Heavy inflammatory cells	0	0	50		0	0	62.5		0	0	62.5	

^*^Significance level at *P* < 0.05; different superscript letters in the same row in each evaluation period are statistically significant different

**Fig. 1:**
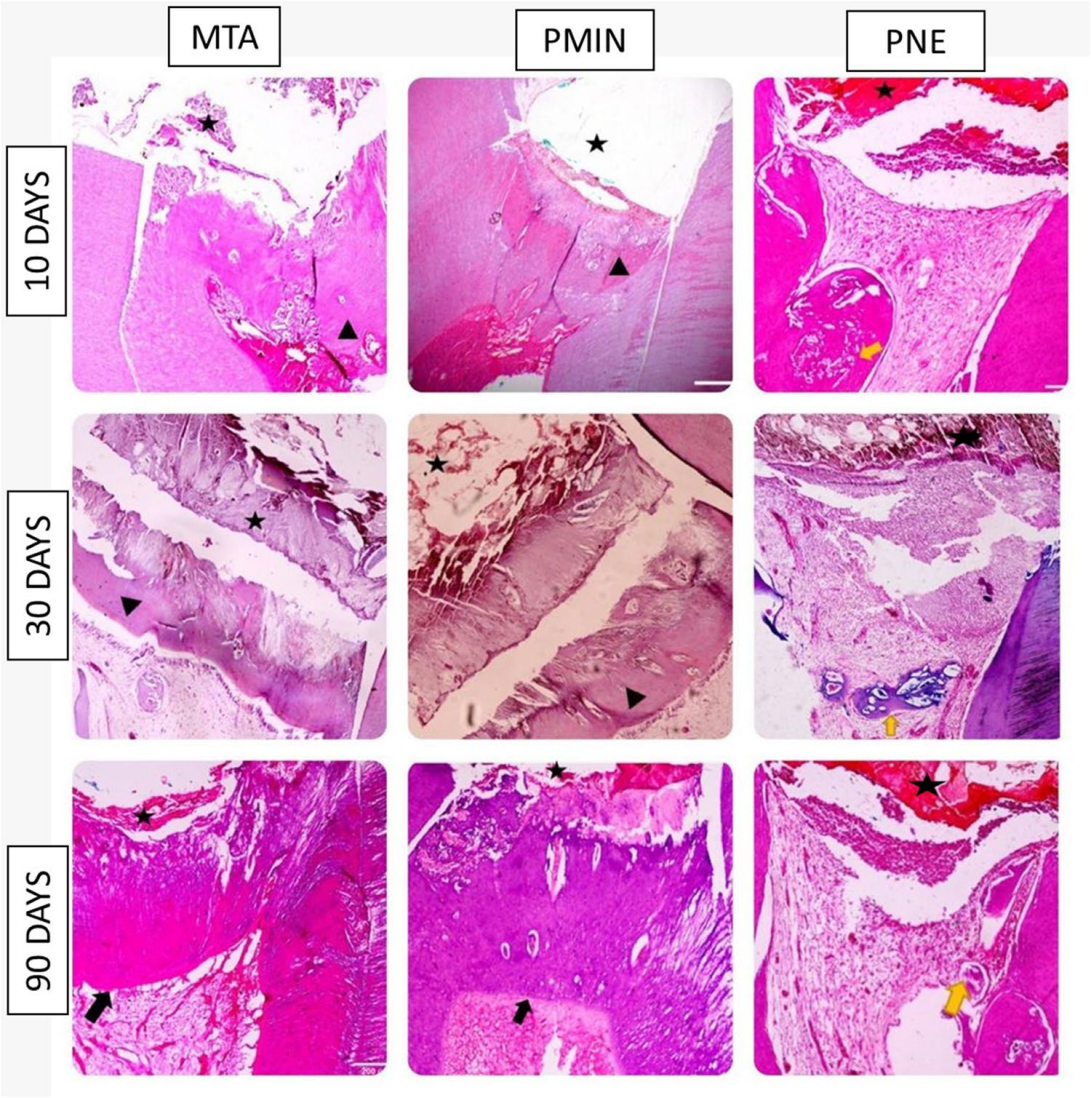
The histologic response of the pulp tissue to the tested biomaterials (black star) (× 10, H&E). Rows represent observation periods (10, 30, and 90 days), and columns represent the tested biomaterials (MTA, PMIN, PNE). An early calcified barrier (black arrow heads). Thick continuous calcific bridge (black arrows). Localized calcific body formed of eosinophilic osteodentin (yellow arrow)

Concerning calcific barrier continuity, a complete bridge was observed in 25% and 50% of the teeth capped either with MTA or PMIN respectively after 10 days, but no bridge was formed in 50% of PNE samples. After 30 and 90 days, both MTA and PMIN subgroups had 100% complete DB, but PNE failed to form a bridge in 37.5% of the samples, 12.5% showed partial DB, while 50% showed initial DB (Fig. 1).

Regarding the evaluation period, there was statistically significant difference with time in MTA and PMIN subgroups (*P* = 0.003 and *P* = 0.048, respectively). While in PNE subgroup, there was no significant difference over time (*P* = 0.845).

Regarding DB thickness after 10 days, 75% of MTA samples had moderate DB thickness, while 25% had thick bridge. Half PMIN samples showed moderate, and the rest showed thick DB. For the PNE subgroup, 50% of the samples showed moderate and thin DB while the rest failed to form DB. After 30 days, both MTA and PMIN showed a thick DB in 87.5% of samples, while PNE showed 12.5% thick DB. After 90 days, MTA and PMIN samples formed a thick DB in 87.5% and 100%, respectively, while PNE samples showed a thin DB in 50% of the samples.

Regarding the observation periods, MTA and PMIN subgroups had statistically significant difference in DB thickness (*P* = 0.009 and *P* = 0.037, respectively), while no significant difference over time was seen in PNE subgroup (*P* = 0.077).

**Fig. 2:**
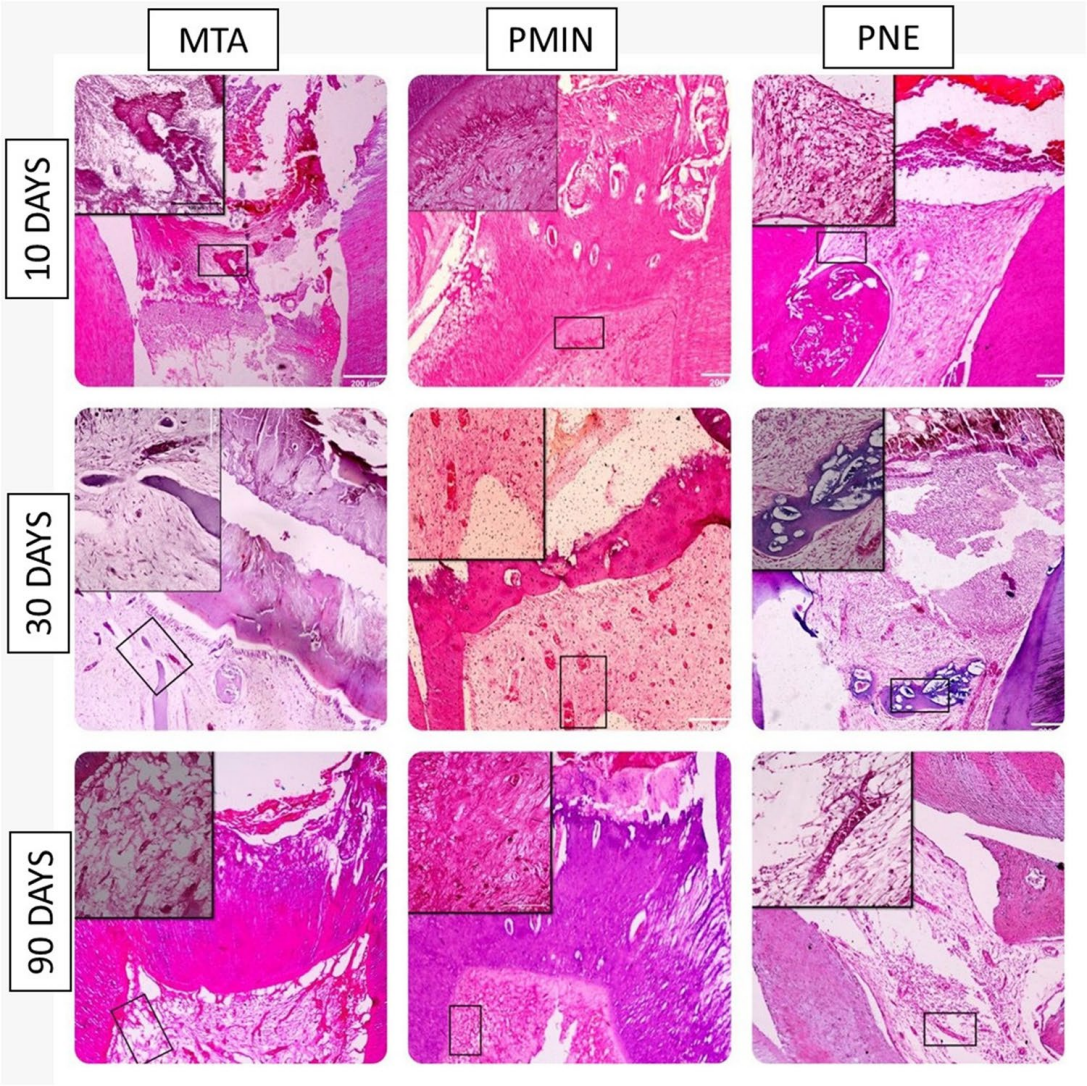
Different inflammatory pulpal responses to the tested biomaterial. Rows represent observation periods (10, 30, and 90 days), and columns represent the tested biomaterials (MTA, PMIN, PNE) (original magnification × 10, × 40, H&E)

Inflammation of the pulp was predominantly absent or mild in 50% of MTA and PMIN samples. This inflammation decreased over time and disappeared in 87.5% of samples at MTA and PMIN subgroups after 90 days with no statistically significant difference either in MTA (*P* = 0.244) or PMIN (*P* = 0.213) over time. On the contrary, unfavorable outcome was reported in the PNE subgroup that exhibited heavy inflammatory cells infiltration in 50% of the samples after 10 days then increased to 62.5% after 90 days and again this had no statistically significant difference over time (*P* = 0.710) (Fig. 2).

### Immunohistochemical findings

**Fig. 3:**
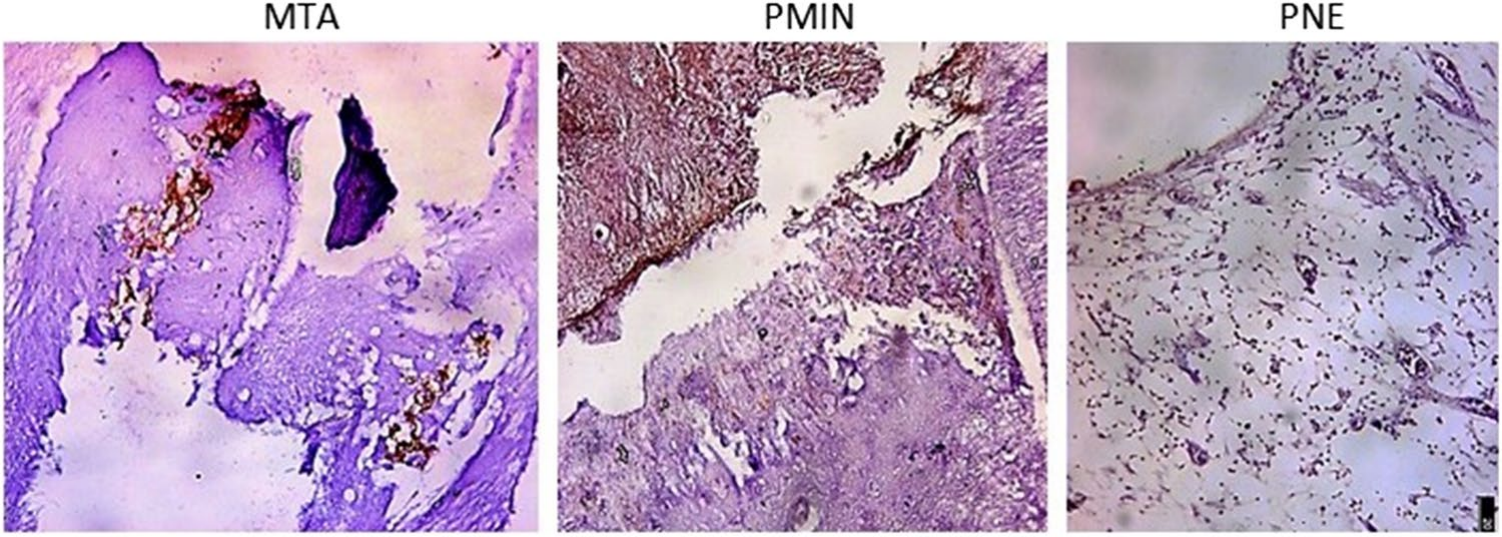
Nuclear and cytoplasmic expression of OPN after 90 days, MTA shows moderate to strong expression, PMIN shows moderate expression, and PNE shows negative results (original magnification × 20)

After 90 days, the MTA subgroup showed the highest mean value of osteopontin positive fraction area (4.660 ± 1.071), followed by the PMIN subgroup (4.130 ± 0.922) without statistically significant differences (*P* > 0.05). The least mean value was recorded in the PNE subgroup (0.5333 ± 0.25166) with statistically significant difference with the remaining subgroups (*P* = 0.002) (Fig. 3).

## Discussion

In vital pulp therapy, it is well understood that the dental pulp must be conserved for a multitude of reasons, including root growth, allowing odontoblasts to form a DB between the capped pulp and the dressing material and maintaining pulp health and function [1, 22]. The ability of two newly introduced vital pulp therapy medicaments materials, PMIN and PNE to create DB after pulpotomy of mechanically deroofing pulps in dogs’ teeth was the subject of this investigation which compared the immunohistopathological behavior of them to the MTA. The null hypothesis was rejected as PNE did not reveal a comparable result to standard MTA in terms of DB formation and inflammatory cell infiltration.

The pulpotomy procedure’s success hinges on preventing bacteria from entering the pulp and controlling bleeding. To prevent microbiological leakage, the teeth were isolated with cotton rolls. Full hemostasis was achieved by applying pressure with a cotton pellet wet with sterile normal saline. Sodium hypochlorite in 0.25% concentrations has also been proven to be beneficial in preventing bleeding without being harmful. However, because sodium hypochlorite has a pH of 12, it has the potential to remove the growth factors from dentin walls [23]. Thus, as the therapeutic action of pulpotomy material was the major subject to be examined, the current investigation was conducted without any additional chemicals or items that could simulate such an action. As a result, only normal saline was administered to reduce the bleeding [24].

Our findings confirmed MTA’s ability to induce a complete DB at 10 days in 25% of the samples, increasing to 100% at 30 and 90 days. The average thickness of this bridge was moderate (0.1–0.25 mm) at 10 days and increased to be thick (> 0.25 mm) at 90 days. This research backs up a prior study that found hard tissue development two weeks following MTA capping [25]. Also, Téclès et al. [26] observed early form of mineralization foci after MTA application in a tooth culture model. Similarly, Tziafas et al. [27], observed irregular osteotypic matrix depositions after pulp capping for 2 weeks in dogs with MTA.

In terms of pulpal inflammation, our data showed that the MTA subgroup had minor to moderate pulpal inflammation at 10 days, which steadily decreased over the evaluation period to almost none at 90 days. These findings are in line with those of other research that has shown that inflammation decreases over time after MTA treatment [25, 28]^.^ Moreover, inflammatory cells were discovered to be almost free after 4 weeks of MTA capping, according to Lee H et al. [29].

PMIN produced a complete DB in 50% of samples after 10 days. At 30, 90 days all samples showed complete DB. The thickness of this bridge was moderate in 50% of the samples, while the rest showed thick DB at 10 days, this progressed to a complete bridge with tubular, thick dentin in all samples at 90 days with almost negligible inflammatory cell infiltrate. These results that beat MTA results can be explained by the presence of propolis in the liquid of PMIN [14]. Previous proofs for the effectiveness of using propolis in vital pulp therapy were presented [10–13]^.^ They stated that it produces consistent outcomes to MTA and appears to be a potential and dependable regenerating medicament. Widjiastuti et al. [11] concluded that the post-administration of propolis extract could enhance odontoblast-like cell and type 1 collagen expression in Wistar rats by stimulating TGF1 synthesis. Propolis is a naturally available biocompatible substance that has multitarget therapeutic features—anti-microbial, anti-inflammatory, and immunomodulatory—resulting from the abundant presence of Flavonoids and Apigenin [30, 31]. The lasts suppress the arachidonic acid lipoxygenase pathway and reduce inflammatory responses while boosting phagocytic activity and enhancing cellular immunity [32]. Moreover, caffeic acid phenethyl ester (CAPE) is one of its active ingredients. CAPE stimulates collagen production and prevents the dental pulp inflammation and degeneration by inhibiting the production of cytokines and chemokines [8, 11].

Adding to the beneficial use of propolis, PMIN powder contains hydroxyapatite crystals (HAP), one of the calcium phosphate biomaterials that have been introduced for hard tissue replacement, bone augmentation, and pulp capping. This may relate to its higher success rate as fibroblasts, in contact with hydroxyapatite, release alkaline phosphatase which is a vital osteoinductive for the differentiation of progenitor cells [33].

At 90 days, PNE failed to promote DB development in 37.5% of the samples; instead, localized calcific area was detected. Only 12.5% of the samples revealed partial moderately thick DB, and 50% revealed initial thin DB with almost heavy infiltration of inflammatory cells. The presence of zinc compounds in PNE powder may account for this unfavorable response as it can be both genotoxic and cytotoxic [34]. Additional analysis revealed that zinc from specific dental materials may contribute to suppressing or slowing the pulpal recovery process by its effect on matrix metalloproteinases (MMPs) [35].

Osteopontin (OPN) is an acidic glycoprotein found in the extracellular matrix that has been linked to a variety of physiological and pathological processes [36]. Many studies deemed OPN a mineralization marker therefore it was used as the immunohistochemistry marker in our 90-day assessment [37, 38] Furthermore, the elevated expression of OPN in reparative dentin was thought to be a sign of the existence of odontoblast cells by Tziafas et al. [39] The results revealed that MTA subgroups have moderate to a strong representation of OPN intervening the hard tissue bridge, which is directly linked to MTA's ability to recruit cells such as fibroblasts and osteoblast-like cells, which are the main stimulants to the expression of genes of hard tissue–related proteins (OPN) [40]. Similarly, at 60 and 90 days, Cosme-Silva et al. [21] noticed that the white MTA-Angelus groups had moderate OPN marker immunolabeling. In the PMIN subgroup, the exposure area was obliterated by a thick, continuous dentin bridge, and the cells displayed nuclear/cytoplasmic positivity resembling that in the MTA subgroup. The majority of PNE samples revealed minimal or no nuclear expression of OPN in spindle-shaped cells, without DB formation, indicating that it was unable to cause pulp cell differentiation and formation of odontoblast-like cells after 90 days in majority of the samples.

One of our study’s limitations is that it was conducted in optimal conditions, with pulps that were healthy when exposed. As a result, the pulp reaction to any material was likely to differ from that of a compromised pulp caused by cavities, cracks, or restoration failure, which allow microorganisms to access the tooth and irritate the pulp. As a result, significant consideration is required to adopt these positive outcomes in clinical settings.

Within the constraints of this research, it can be concluded that the histologic outcomes of pulpotomy with PMIN are equivalent to those of MTA, making PMIN a viable alternative. To corroborate these findings, more prospective clinical investigations should be done.

## Data Availability

Both are available upon request.
